# The burden of hospitalized sports-related injuries in children: an Australian population-based study, 2005–2013

**DOI:** 10.1186/s40621-018-0175-6

**Published:** 2018-12-17

**Authors:** Francisco J. Schneuer, Jane C. Bell, Susan E. Adams, Julie Brown, Caroline Finch, Natasha Nassar

**Affiliations:** 1 Child Population and Translational Health Research, The Children’s Hospital at Westmead Clinical School, Level 2 The Hub, Charles Perkins Centre D17, The University of Sydney, Westmead, NSW 2006 Australia; 2 Injury Prevention, Neuroscience Research Australia, Randwick, NSW Australia; 3 Department Paediatric Surgery, Sydney Children’s Hospital, Randwick, NSW Australia; 4 School of Women’s and Children’s Health, University of New South Wales, Kensington, NSW Australia; 5 Australian Centre for Research into Injury in Sport and its Prevention (ACRISP), Edith Cowan University, Perth, WA Australia

**Keywords:** Sports-related injuries, Children and adolescent sports, Epidemiology, Hospital admissions, Traumatic brain injury

## Abstract

**Background:**

There is concern about recent increase and severity of sports-related injuries in children. Despite the benefits of sports participation, injuries may carry long-term health consequences. We aimed to evaluate the prevalence, characteristics and types of hospitalized sports-related injuries in children.

**Methods:**

Population-based study of all acute sports-related injuries requiring hospitalization in children 5 to 15 years of age in New South Wales (NSW), Australia, 2005–2013. Health information was obtained from the NSW Admitted Patient Data Collection, a census of all hospital admissions from public and private hospitals. Children with a recorded ICD10-AM injury code (S00-T79) and sport-related activity code (U50-U70) were included. Prevalence and trend in injuries by age group, sporting code, body region affected and type of injury were assessed.

**Results:**

There was a total of 20,034 hospitalizations for sports-related injuries (2.7% of all hospitalizations in children aged 5–15 years), involving 21,346 recorded injuries in 19,576 children. The overall population hospitalization period prevalence was 227 per 100,000 children aged 5–15 years in 2005–2013, remaining stable over time (RR 0.99; 95% CI 0.98–1.00). Football codes such as rugby league/union and soccer combined represented nearly two thirds of the total (60%). The most common body regions affected were the forearm (31%) head (15%) and hand injuries (13%). Fractures accounted for 65% of injuries followed by dislocations (10%) and traumatic brain injury (10%). Compared to other age groups, children aged 5–8 years had double the proportion of shoulder (15% vs. 7%) while 13–15 year olds had higher proportion of lower-leg (14% vs. 8%) and knee (6% vs.2%) injuries. One in seven injuries sustained while playing rugby league/union, baseball and hockey were traumatic brain injuries. A total of 444 (2.2%) of children had more than one hospitalization for sports-related injuries.

**Conclusion:**

On average, six children were hospitalized every day for sports-related injuries in the last decade with trends remaining stable. The most common sports involved were football codes, one in three injuries involved the forearm and two thirds were fractures. These findings can be used to inform health policy and sporting governing bodies to target preventive interventions and promote safe sports participation in children.

**Electronic supplementary material:**

The online version of this article (10.1186/s40621-018-0175-6) contains supplementary material, which is available to authorized users.

## Background

Sports participation has multiple benefits on children’s psychological, physical and social capabilities (Eime et al., 2013); however, with recent increases in sports participation (Eime et al., 2016), injuries have become an important pediatric public health issue. Population-based studies have reported that yearly between 3% and 8% of children experience sports-related injuries (Bruhmann & Schneider, 2011; Sheu et al., 2016), and recent studies have indicated that sport-related injuries represent one third of all emergency department (ED) visits among school-age children (Harmon et al., 2018) and occur five times more frequently than those caused by injuries from road accidents (Finch et al., 2014). They are also the second most common cause of severe injuries such as traumatic brain or spinal cord in children over 10 years of age (Oliver et al., 2018). Increasing trends reported for traumatic brain (Bayt & Bell, 2016), internal organs (Bayt & Bell, 2016), severe knee (Shaw & Finch, 2017) and dental (James et al., 2018) injuries sustained by children while playing sports in the last decade have raised concerns among parents and pediatric health professionals in relation to their potential long-term consequences, particularly of severe injuries. This has led to the call for better reporting and monitoring of active recreation and sports-related injuries in children as well as increasing preventive interventions and reducing harmful contact (Finch et al., 2014; Pollock et al., 2017).

In Australia, there has been little exploration of the burden of this problem in pediatric healthcare. The latest Australian report on sports-related injury hospitalizations in 2012 described a prevalence of 6.6 per 1000 children aged 15 to 17 years but no information was reported for children aged below 15 years (Australian Institute of Health and Welfare, 2014). Another study reported a sustained increase of sports-related injury hospitalizations and ED visits for children in the last decade (Finch et al., 2014), however, information on sport-specific characteristics and patterns of injuries are lacking. Other studies have been limited by small numbers, and included only one sport or one type of injury (Shaw & Finch, 2017; Crowe et al., 2010; Leung et al., 2017). Population-level information on the burden of sports-related injuries is essential to define the scope of this healthcare issue, provide key indicators to direct the implementation and evaluation of preventive strategies and the development of relevant pediatric health policy.

The aim of the study was to investigate sports-related injuries requiring hospitalization in children by evaluating trends, characteristics and patterns of occurrence.

## Methods

We included all hospitalizations for sports-related injuries in children aged 5 to 15 years of age treated in New South Wales (NSW), Australia, between 2005 and 2013 inclusive. Health information was obtained from the NSW Admitted Patient Data Collection (APDC). The APDC is a census of all in-patient hospital admissions from public and private hospitals that includes patient’s demographic information and all diagnosis and procedures for each admission. In the APDC, diagnoses are coded according to the 10th revision of the International Classification of Diseases, Australian Modification (ICD-10-AM) and procedures coded using the Australian Classification of Health Interventions (ACHI).

Injuries sustained while playing sports or during active recreation were identified using the ICD-10-AM external causes of morbidity and mortality or activity codes (U50-U71) that ascertain the relevant causal sport for injury-related hospital admissions. Some sports with similar features such as rugby league/rugby union and martial arts/wrestling were combined. We excluded injuries sustained while involved in wheeled recreation activities (e.g. bike riding, skateboarding, scooter riding and roller blading), using playground equipment (e.g. trampoline, flying fox), motorized land, water or aero sports, firearm shooting sports and those caused by anaphylactic shock or by animal encounters (e.g. dog bites or sharks) because they were unlikely to occur in an organized sporting activity.

Injuries were defined by body region (head, eye, neck, shoulders, thorax, forearm, hand, abdomen, hip or thigh, knee, lower-leg and ankle or foot) and type of injury (superficial, open wound, fractures, dislocations, muscle or joints, dental, nerve or spinal cord, traumatic brain injury (TBI), ocular, internal organ, foreign body and drowning) following the Australian Sports Injury Data Dictionary (SportSafe Australia, 1999). To apply consistency and reliability in our ascertainment of TBI we used the ICD-10-AM codes identified in a recent review by Chan et al. (Chan et al., 2015). Other injury factors investigated included place of occurrence (Y92: home, school, sports facility, public place or road, outdoors or farms and unspecified), day of injury (weekday/weekend), and length of hospital stay (LOS) in days as a proxy for severity. LOS was categorized according to percentiles into 1 day (<75th), 2 days (75th–90th) and 3 or more days (>90th).

Demographic characteristics included sex, age group (5–8, 9–12 and 13–15 years of age) and patient residence (major city, inner regional, outer regional/remote). Patient residence was categorized according to the Australian Bureau of Statistics (ABS) Statistical Geography Standard using patient residential postcodes (Australian Bureu of Statistics, 2011).

As LOS and health resource use including major surgery, intensive care unit, mechanical ventilation or blood transfusion have been previously reported as good predictors of injury severity (Newgard et al., 2010), severe injuries were identified as those requiring hospital admission with LOS 3+ days and/or those requiring these interventions. We also identified subsequent sports-related injuries requiring admissions distinct to an initial sports-related hospitalization. To avoid double counting injuries from re-admissions or transfers to other hospitals with the same injury, we excluded subsequent admissions occurring within 30 days of the initial admission and with the same recorded diagnosis.

### Statistical analysis

The overall population period prevalence of sports-related injury hospitalizations per 100,000 children was calculated using NSW population data obtained from the ABS. It was analyzed by sex and broad age groups. We used negative binomial regression (relative risk (RR) with 95% CI) to evaluate changes in population prevalence overtime, including the log of the population as offset and stratified by age groups. We also calculated proportions of hospitalizations by location of occurrence and day of the week out of total sports-related hospitalizations. The proportion of hospitalizations with severe injuries by sporting code was also calculated. As some hospitalizations included multiple injuries we used total injuries to calculate the proportion of injuries by sporting code, age groups, body region affected, type of injury and subsequent injuries. Injuries occurring in different body regions were counted separately. Rates of subsequent hospitalized sports related-injuries were evaluated by the sporting code of the initial admission, body region and type of injury. All analyses were conducted using SAS, 9.4 (SAS Institute, Cary, NC, USA).

## Results

**Fig. 1:**
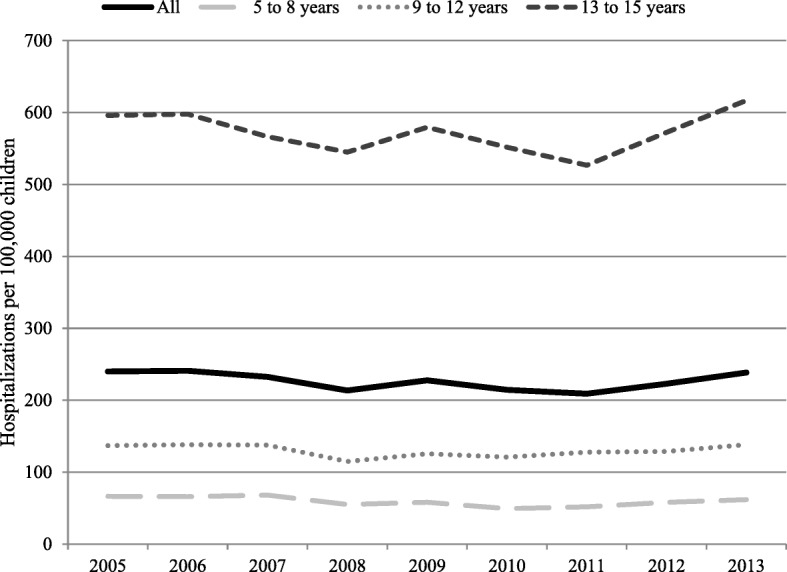
Trend of sports-related injuries hospitalizations by age groups in children in NSW, Australia, 2005–2013. Relative risk RR (95% CI) for trend in population prevalence: all RR 0.99 (0.98–1.00); 5 to 8 years RR 0.98 (0.96–1.00); 9 to 12 years RR 1.00 (0.98–1.00) and 13 to 15 years: RR 1.00 (0.99–1.01)

There was a total of 20,034 hospitalizations for sports-related injuries in NSW in 2005–2013, representing 2.7% of all hospitalizations in children aged 5–15 years. Hospitalizations included 21,346 injuries in 19,576 children. Multiple injuries were recorded in 1210 (6%) hospitalizations, with a median (range) of 2 (2–6). The child characteristics of all hospitalizations are presented in Table 1. Four fifths of hospitalizations occurred in males, over two thirds in 13–15 year olds and three quarters from injuries sustained in sporting facilities. The overall population period prevalence of hospitalizations was 227 per 100,000 children aged 5–15 years in 2005–2013, averaging six hospitalizations per day during the study period. The prevalence increased with age from 59 per 100,000 in children 5–8 years to 573 in the 13–15 years group. The prevalence of all sports-related hospitalizations remained stable overtime (RR 0.99; 95% CI 0.98–1.00), and for all age groups (Fig. 1). The prevalence in males (357 per 100,000) was over three times higher than for females (87 per 100,000). One third of hospitalizations occurred during weekends and over two thirds had LOS of one day (Table 1).

**Table 1:** Demographic characteristics of children’s hospitalizations for sports-related injuries in NSW, Australia, 2005–2013

All	N = 20,034 n (%)
Sex	
Male	16,260 (81.2)
Female	3774 (18.8)
Age	
5–8 years	1900 (9.5)
9–12 years	4158 (20.8)
13–15 years	13,976 (69.8)
Patient residence^a^	
Major Cities	14,140 (70.8)
Inner Regional	4304 (21.6)
Outer regional / remote	1517 (7.6)
Injury location	
Sports facility or area	15,621 (78.0)
School	2601 (13.0)
Farm or outdoors area	702 (3.5)
Unspecified place	455 (2.3)
Home	346 (1.7)
Public place, road or highway	309 (1.5)
Day of occurrence	
Weekday	13,244 (66.1)
Weekend	6790 (33.9)
Length of hospital stay (days)	
1	14,376 (71.8)
2	4508 (22.5)
3+	1150 (5.7)

^a^percentages do not add to 100 due to missing values

**Fig. 2:**
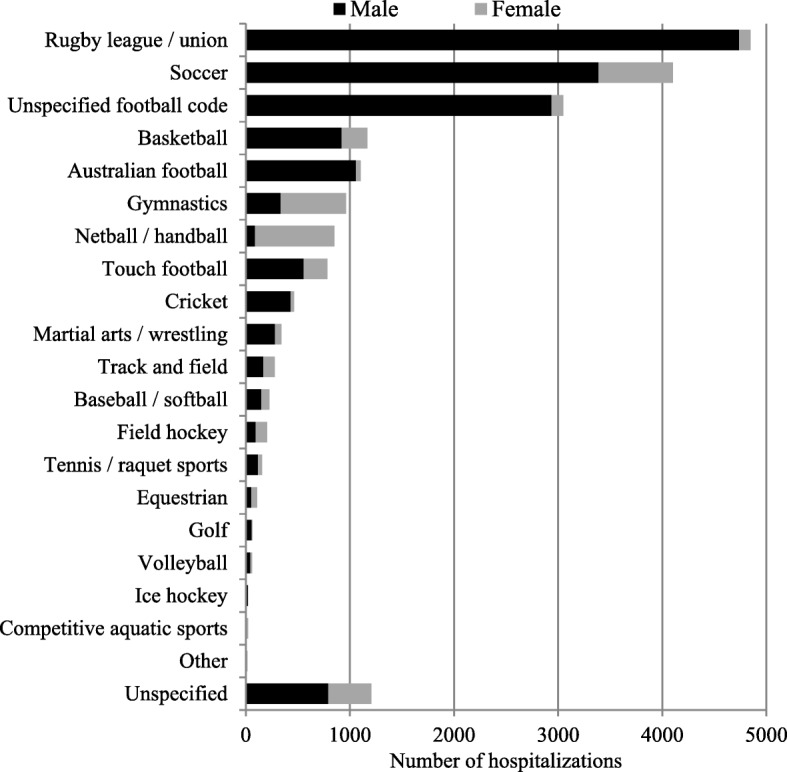
Sports-related injuries hospitalizations by sex in children in NSW, Australia, 2005–2013

**Fig. 3:**
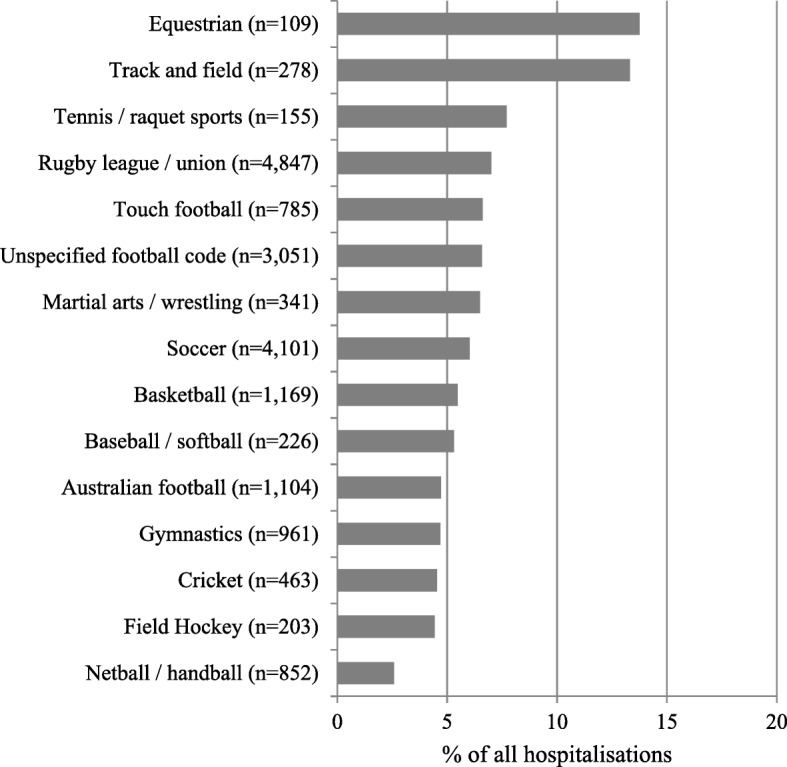
Sports-related injury hospitalizations of severe injuries by sporting code in children in NSW, Australia, 2005–2013. Severe injuries represent those with length of stay of 3+ days, requiring major surgery, intensive care unit admission, mechanical ventilation or blood transfusion; Only sports with >100 total hospitalizations are reported

Figure 2 presents the number of hospitalization for sports-related injuries by sex and sporting code. Football codes such as rugby league/union and soccer combined represented nearly two thirds of the total (60%). For most sports, hospitalizations were more frequent in males, while hospitalizations were more common in females in some sports such as netball (89%) and gymnastics (65%). In a majority of sports, hospitalizations were more common for children aged 13–15 years and accounted for up to 80% of hospitalizations for some sports like field hockey, Australian football and rugby league/union. However, in sports such as gymnastics and track and field, an equal proportion of children aged < 12 were hospitalized compared to older children (Additional file 1: Figure S1). There were a total of 1186 (5.9%) hospitalizations with severe injuries, ranging from 14% for equestrian sports (*n* = 109) to 2.0% for netball (*n* = 852) (Fig. 3). There was a slight increase in the proportion of severe injuries with increasing age from 4.7% in the 5–8 years group to 6.3% in 13–15 year olds.

When evaluating total injuries overall, one in three injuries affected the forearm (31%), followed by the head (15%), hand (13%) and lower-leg (12%) (Table 2). Compared to other age groups, children aged 5–8 years had double the proportion of shoulder injuries; while a lower proportion had a hand and neck injury. Injuries to the abdomen, knee and lower-leg increased in proportion with age; while a lower proportion of children aged 13–15 years had forearm injuries compared with younger age groups. The most common type of injury were fractures representing over half of the total (65%) followed by dislocations, (10%) and TBI (10%) (Table 3). The proportion of dislocations and TBI increased with age and internal organs injuries were more common in children aged 13–15 years. In contrast, the proportion of fractures and open wounds decreased with age and children aged 5–8 years sustained less muscle or joints injuries.

**Table 2:** Hospitalized sports-related injuries by body region among children aged 5 to 15 years in NSW, Australia, 2005–2013

Body region of injury	All N = 21,346^a^ *n* (%)^b^	5–8 years N = 2,015^a^ *n* (%)^b^	9–12 years N = 4,426^a^ *n* (%)^b^	13–15 years N = 14,905^a^ *n* (%)^b^
Forearm	6701 (31.4)	849 (42.1)	1790 (40.4)	4062 (27.3)
Head	3223 (15.1)	269 (13.3)	620 (14.0)	2334 (15.7)
Hand	2774 (13.0)	162 (8.0)	575 (13.0)	2037 (13.7)
Lower-leg	2659 (12.5)	151 (7.5)	398 (9.0)	2110 (14.2)
Shoulder	1641 (7.7)	305 (15.1)	255 (5.8)	1081 (7.3)
Neck	1006 (4.7)	50 (2.5)	240 (5.4)	716 (4.8)
Knee	982 (4.6)	26 (1.3)	87 (2.0)	869 (5.8)
Abdomen	597 (2.8)	32 (1.6)	106 (2.4)	459 (3.1)
Hip or thigh	573 (2.7)	59 (2.9)	120 (2.7)	394 (2.6)
Ankle or foot	484 (2.3)	37 (1.8)	82 (1.9)	365 (2.4)
Thorax	205 (1.0)	12 (0.6)	33 (0.7)	160 (1.1)
Eye	155 (0.7)	21 (1.0)	35 (0.8)	99 (0.7)

^a^Represents total recorded injuries (one hospitalization can include multiple injuries)

^b^Percentages do not add to 100 due to ~2% recorded unspecified body region (not presented)

**Table 3:** Types of hospitalized sports-related injuries among children aged 5 to 15 years in NSW, Australia, 2005–2013

Type of injury	All N = 21,346^a^ *n* (%)^b^	5–8 years N = 2,015^a^ *n* (%)^b^	9–12 years N = 4,426^a^ *n* (%)^b^	13–15 years N = 14,905^a^ *n* (%)^b^
Fractures	13,781 (64.6)	1412 (70.1)	2920 (66.0)	9449 (63.4)
Dislocations	2228 (10.4)	102 (5.1)	332 (7.5)	1794 (12)
Traumatic brain injury	2173 (10.2)	156 (7.7)	406 (9.2)	1611 (10.8)
Open wounds	1088 (5.1)	183 (9.1)	272 (6.1)	633 (4.2)
Superficial	835 (3.9)	81 (4.0)	195 (4.4)	559 (3.8)
Muscle or joints	767 (3.6)	47 (2.3)	154 (3.5)	566 (3.8)
Internal organ	256 (1.2)	12 (0.6)	30 (0.7)	214 (1.4)
Ocular	189 (0.9)	18 (0.9)	37 (0.8)	134 (0.9)
Nerve or spinal cord	104 (0.5)	11 (0.5)	20 (0.5)	73 (0.5)
Dental	50 (0.2)	8 (0.4)	16 (0.4)	26 (0.2)
Blood vessel	13 (0.1)	–	–	11 (0.1)
Foreign body	5 (< 0.1)	–	–	–

^a^Represents total recorded injuries (one hospitalization may include multiple injuries)

^b^Percentages do not add to 100 due to recorded unspecified type (not presented); − indicate <5 injuries

**Fig. 4:**
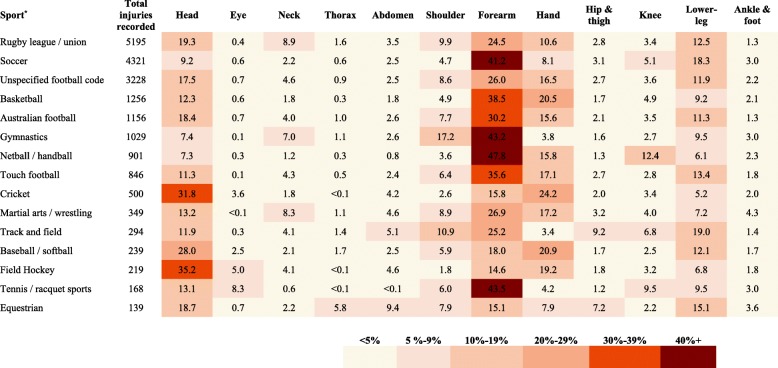
Proportion of hospitalized sports-related injuries by sport and body region in children in NSW, Australia, 2005–2013. ^*^Only sports with >100 total hospitalizations are reported; numbers represent percentages

The proportion of injuries by sporting code and body region is presented in Fig. 4. Forearm injuries were the most common type in a majority of sports with at least 25% occurring in 10 out of 15 sports. Injuries to the head were also common and particularly high (~30%) in sports with a fast travelling hard ball such as cricket, field hockey and baseball. One in five injuries of children playing cricket, basketball or baseball involved the hand; followed by lower-leg injuries. Other body regions were less frequently injured across a majority of sports. However, exceptions included higher shoulder injuries for gymnastics (17%); knee injuries for netball (12%) and abdomen injuries for equestrian sports (9%).

**Fig. 5:**
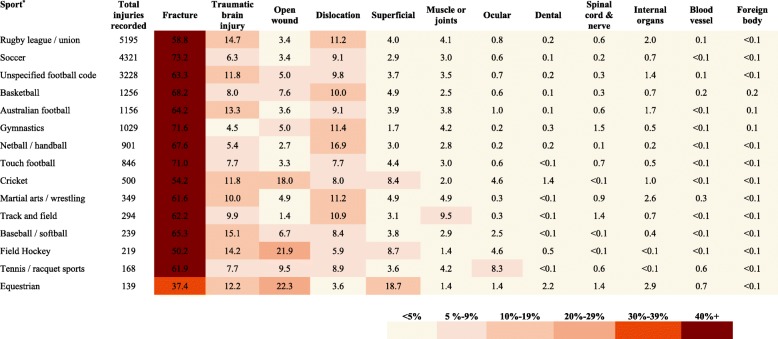
Proportion of hospitalized sports-related injuries by sport and type of injury in children in NSW, Australia, 2005–2013. ^*^Only sports with >100 total hospitalizations are reported; numbers represent percentages

Figure 5 presents the type of injury by sporting code. The most common type of injury within individual sports was a fracture with over 70% for soccer, gymnastics and touch football. Dislocations were particularly higher in netball with 17%. Approximately one in seven injuries in children participating in rugby league/union, baseball/softball and field hockey were TBI. Some sports such as equestrian, field hockey and cricket had a higher frequency of open wounds and superficial injuries (>18%). The proportion of muscle, joint and ocular injuries was more than double compared to the average of all sports for track and field (10%) and tennis/racquet sports (8%), respectively. Other types of injuries were consistently less frequent across all sporting codes.

Overall, 444 (2.2%) of children had a subsequent hospitalization for sports-related injuries, distinct to the initial admission. After evaluating subsequent sports-related hospitalizations by the initial sporting code, body region or type of injury, only a small proportion had subsequent hospitalizations with little difference across groups (Additional file 2: Table S1).

## Discussion

In this population-based study, we found that hospitalizations for sport-related injuries in children were stable in the last decade, but still represent a frequent event impacting children’s health. Males outnumbered females in rates of injuries in most sports while important contributors were football codes such as rugby and Australian football. One in three injuries affected the forearm and over half were fractures followed by dislocations and TBI. The body region affected, the type of injury and the sporting code varied by the children’s age. While a majority were one-day hospitalizations (72%), importantly, severe injuries requiring lengthy hospital stay (three or more days) were not infrequent and as high as one in seven in some sports.

We found a stable prevalence of hospitalizations for sports-related injuries in the last decade; however, hospitalizations may represent the more severe spectrum of injuries and timeline assessment of total sports injuries may provide different results depending on where they were treated and the local healthcare system. In Australia, a recent study from the state of Victoria using statewide hospitalization data found that sports-related injuries increased by almost 30% in the past decade (4% annual increase) (Finch et al., 2014), compared to decreasing road traffic injuries. The authors also found that sports-related injuries costed on average 8.5 million dollars per year to the healthcare system. The main difference with our study is that the Victorian study included information on injuries presenting to emergency department that were the main driver (three quarters of the total) of the increasing trend reported. Findings from the United States and Canada have also found increases in ED presentations for all sports injuries (Bayt & Bell, 2016), TBI (Coronado et al., 2015), and in injuries caused by playing different sports such as track and field, soccer and American football in the last decade (Lykissas et al., 2013; Reid et al., 2012). In contrast, injuries from other popular recreational activities have decreased overtime (Lykissas et al., 2013). Moreover, another study from the Netherlands reported that sports-related injuries represented the highest increase in incidence rates (5.4% annually) of all pediatric injury-related hospitalizations between 2001 and 2009 (Janssens et al., 2014). Population level evaluation has been possible in some countries because of the existence of local surveillance systems such as the United States National Electronic Injury Surveillance System (Quinlan et al., 1999) and Canadian Hospitals Injury Reporting and Prevention Program (Mackenzie & Pless, 1999). This is of particular relevance because comparison of results may be difficult due to significant variability in sporting participation, culture and popularity among countries. This has been highlighted in studies from nationwide surveys of 11–15 year old students reporting differences ranging from 6% and 27% of yearly rates of sports-related injury that required medical attention in children from low-middle income countries (25 countries) and between 23% and 64% in high income countries (35 countries) (Street & Jacobsen, 2017; Pickett et al., 2005). Although, we evaluated this issue in the tertiary healthcare system using state wide administrative heath data which provide important information for health policy and relevant sporting bodies, there is no formal surveillance system for pediatric injuries in Australia. An important step forward has been the recent publication of the first Child Safety Good Practice Guide which has provided key recommendations for child injury prevention including those specific to sports-related injuries such as improving the play environment and rules, the use of safety gear and incorporating preventive protocols in training (Adams et al., 2016). However, without improving the standards and coverage of sports-injury surveillance information in children, under reporting and poor quality of data remains a setback for guiding preventive strategies, clinical decision making and post-injury management (Finch & Fortington, 2018).

We found that fractures were the most common type of injuries requiring hospitalization with an overall rate of 55%. Other population-based studies have reported between 18% and 30% injuries presenting to ED were fractures (Fridman et al., 2013; Howard et al., 2014), however, the lower proportion may be the result of the lower severity of injuries presenting to ED not requiring hospitalization. The decrease in the proportion of fractures with increasing age has also been reported elsewhere (Stracciolini et al., 2013), and may be attributed to an increase in overuse musculoskeletal injuries in older children or because adolescents are predominantly treated in ED or as outpatients rather than being admitted. Increased focus on preventive programs to reduce impact injuries including strength training, rule changes, reducing/removing body contact and protective equipment are important.

Although TBI represented a low proportion of total injuries in our data, one in four TBIs requiring hospitalization in children in NSW are sports-related (Amaranath et al., 2014) and therefore are an area of special interest in prevention of sports injuries due to potential long-term consequences. We found that among team sports, rugby codes had the highest rates of severe and TBIs combined with the highest volume of injuries. A recent systematic review and meta-analysis reported that rugby had the highest incidence of concussion among competitive sports (Pfister et al., 2016), while another review found that youth rugby players had a 23% risk of concussion over a single season (Kirkwood et al., 2015). This has resulted in an increasing body of evidence supporting a cautionary approach by removing the tackle from youth rugby in the early stages of rugby participation (Pollock et al., 2017). Also concerning is the poor adherence to guidelines for returning to play following concussion, with a cohort study reporting that 95% percent of schoolboy rugby union players return to training and play before the recommended timeline of three weeks (Hollis et al., 2012). Future research is needed to elucidate the long-term outcomes of TBI and other severe injuries such as anterior cruciate ligament tears requiring reconstruction and physeal fractures (growth disorders).

We did not find significant differences in subsequent hospitalization for sports-related injuries among sporting codes, body region and type of injuries. In contrast, a previous study reported that having a knee injury increased the risk of subsequent lower limb injuries (Toohey et al., 2017); and another study found increased injuries in children following concussion when returning to play (Brooks et al., 2016). These inconsistencies may require further exploration particularly by evaluating all types of sports injuries and not only those treated in hospitals.

We have shown that pediatric sports injuries requiring hospitalization affect six children daily and; given they can be prevented (Lauersen et al., 2014), there is substantial incentive to introduce and promote preventive health policy and interventions to reduce their occurrence. A meta-analysis evaluating interventions to reduce sports-related injuries in children, reported a significant reduction in sports-related injuries (overall RR 0.54; 95% CI 0.45–0.67) (Rossler et al., 2014), particularly interventions based on resistance training (plyometrics) and jumping exercises. Accordingly, resistance training has been widely recommended as early as age six years due to its benefits in increasing muscular strength, speed, balance and flexibility among other benefits that are crucial to prevent injuries (Faude et al., 2017). Additionally, in those contact sports that apply restrictions to contact initiation in children, a comprehensive coach and athlete education program to promote the adherence to guidelines reduces the rates of injuries (Kerr et al., 2015). Preventive screening examinations performed by qualified clinicians such as the preparticipation physical evaluation may also reduce injury rates by identifying children with potential abnormal physical factors that increase their risk; provide recommendations or protectively disqualify children to participate in particular sports (Lehman & Carl, 2017). Underutilization of protective gear may be relevant for some sports like cricket or equestrian sports is also an important target for preventive efforts (Short et al., 2018).

The main strength of the study is the large nature and coverage of our study by using statewide administrative health data. Using hospital data also ensures the reporting of all injuries sustained avoiding the bias of other studies based on surveys reporting only the most serious injury (Gupta et al., 2016). One limitation of the study is that we did not have information on emergency department and primary care presentations, and thus the results from the study represent a partial picture of the total burden of sports-related injuries in children. Nevertheless, given hospitalizations represent the more severe spectrum of total injuries and thus the results reported here should be interpreted in this context. Another limitation is that our prevalence of sports-related injury hospitalizations may be underestimated due to missing information on activity code in hospital data (30% of total injury-related hospitalizations) (Finch & Boufous, 2008), while hospitalizations attributed to specific sporting codes may also be underestimated due to those with a recorded unspecified sport (6%). Additionally, without reliable data on exposure or participation, it was not possible to determine a more accurate estimation on the incidence of sports injuries in children and comparison between sports.

## Conclusions

Sports-related injuries requiring hospitalization are common, with six children hospitalized every day in the last decade, with the trend remaining stable. Males were four times more likely to be hospitalized for sports related injuries, one in three injuries involved the forearm and two thirds were fractures. These findings will inform health policy and sporting governing bodies of specific areas to target preventive interventions to decrease the burden of sports-related injuries and promote safe sports participation to ensure optimal health and wellbeing of children.

## Additional files

Additional file 1: **Figure S1.** Sports-related injuries hospitalizations by sport and age group in children in NSW, Australia, 2005–2013. Only sports with > 100 total hospitalizations are reported. (DOCX 15 kb)

Additional file 2: **Table S1.** Proportion of subsequent hospitalized sports-related injuries by sporting code, type and body region in children aged 5–15 years in NSW, Australia, 2005–2013. (DOCX 17 kb)

## Abbreviations

ABS: Australian Bureau of Statistics
ACHI: Australian Classification of Health Interventions
APDC: Admitted Patient Data Collection
ED: emergency department
ICD-10-AM: International Classification of Diseases, Australian Modification
LOS: length of hospital stay
RR: relative risk
TBI: traumatic brain injury
