# Supplementary material for: The burden of hospitalized sports-related injuries in children: an Australian population-based study, 2005–2013
Source: Inj Epidemiol. 2018 Dec 17;5:45. doi: 10.1186/s40621-018-0175-6 (PMC6295440; doi:10.1186/s40621-018-0175-6)
Supplement: Supplementary file 2 — Table S1. Proportion of subsequent hospitalized sports-related injuries by sporting code, type and body region in children aged 5–15 years in NSW, Australia, 2005–2013. (DOCX 17 kb) [file 40621_2018_175_MOESM2_ESM.docx]

**Table S1:** Proportion of subsequent hospitalized sports-related injuries by sporting code, type and body region in children aged 5-15 years in NSW, Australia, 2005-2013

| **Sporting code** | **Initial hospitalizations** | **Subsequent hospitalizations^*^** | **%** |
| --- | --- | --- | --- |
| Rugby league / union | 4703 | 138 | 2.9 |
| Soccer | 4018 | 74 | 1.8 |
| Unspecified football code | 2967 | 97 | 3.3 |
| Basketball | 1144 | 18 | 1.6 |
| Australian football | 1073 | 28 | 2.6 |
| Gymnastics | 949 | 15 | 1.6 |
| Netball / handball | 842 | 7 | 0.8 |
| Touch football | 771 | 13 | 1.7 |
| Cricket | 456 | 13 | 2.9 |
| Martial arts / wrestling | 334 | 5 | 1.5 |
| Track and field | 274 | 7 | 2.6 |
| Baseball / softball | 222 | - | - |
| Field Hockey | 202 | - | - |
| Tennis / racquet sports | 153 | - | - |
| Equestrian | 108 | - | - |
| **Body region** | **Injuries recorded in initial hospitalization** | **Subsequent hospitalizations** | **%** |
| Forearm | 6586 | 145 | 2.2 |
| Head | 3128 | 79 | 2.5 |
| Hand | 2716 | 58 | 2.1 |
| Lower-leg | 2592 | 50 | 1.9 |
| Shoulder | 1604 | 32 | 2.0 |
| Neck | 978 | 30 | 3.1 |
| Knee | 950 | 14 | 1.5 |
| Abdomen | 578 | 20 | 3.5 |
| Hip or thigh | 555 | 17 | 3.1 |
| Ankle or foot | 468 | 8 | 1.7 |
| Thorax | 199 | 10 | 5.0 |
| Eye | 153 | 6 | 3.9 |
| **Type of injury** |  |  |  |
| Fracture | 13507 | 263 | 1.9 |
| Dislocation | 2151 | 55 | 2.6 |
| Traumatic brain injury | 2105 | 60 | 2.9 |
| Open wound | 1066 | 26 | 2.4 |
| Superficial | 809 | 23 | 2.8 |
| Muscle and joints | 741 | 22 | 3.0 |
| Internal organs | 248 | 13 | 5.2 |
| Ocular | 187 | 7 | 3.7 |

^*^For any sports-related injury
