# Supplementary material for: The burden of hospitalized sports-related injuries in children: an Australian population-based study, 2005–2013
Source: Inj Epidemiol. 2018 Dec 17;5:45. doi: 10.1186/s40621-018-0175-6 (PMC6295440; doi:10.1186/s40621-018-0175-6)
Supplement: Supplementary file 2 [file 40621_2018_175_MOESM1_ESM.docx]

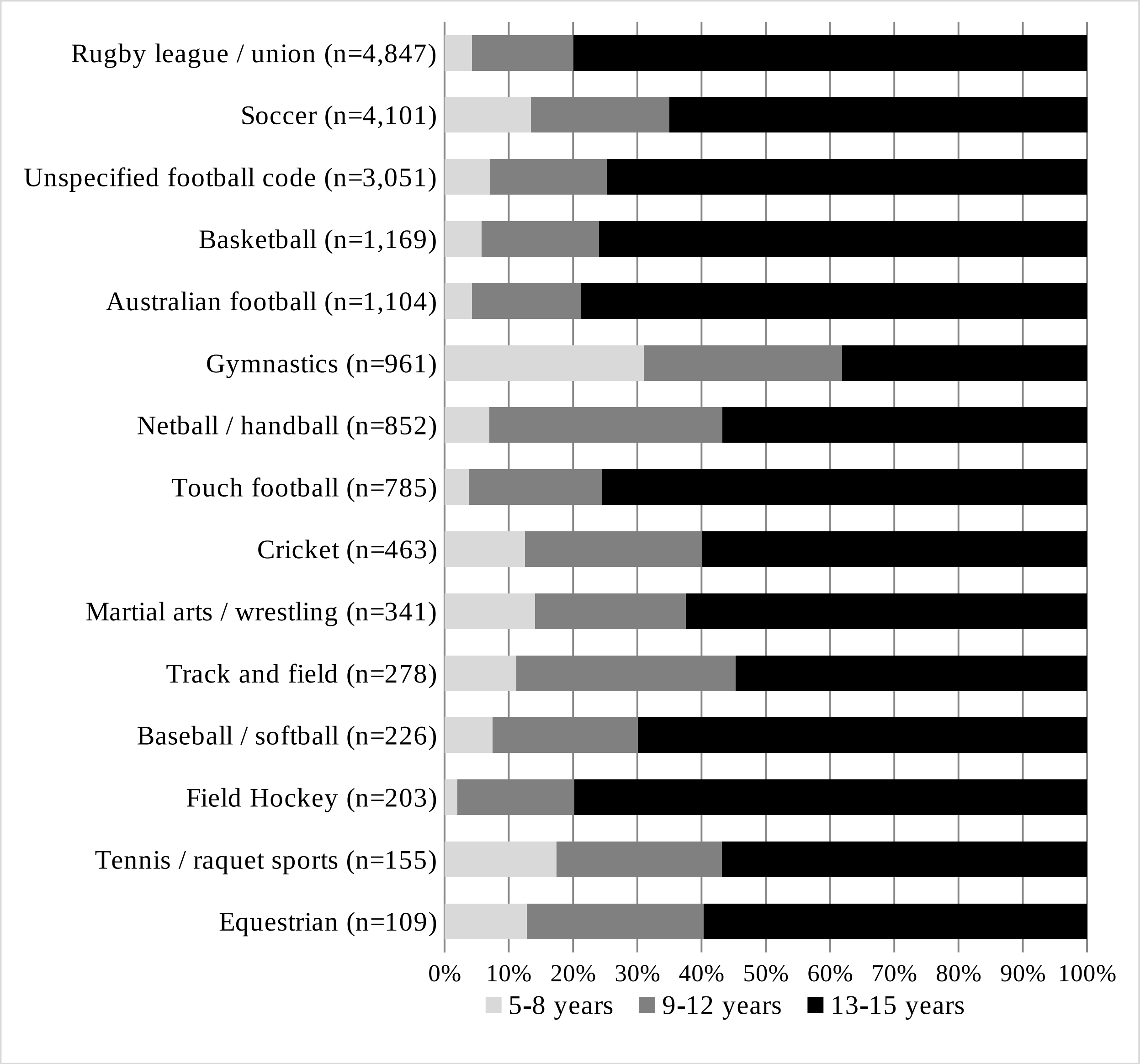

Rugby league / union (n=4,847)
Soccer (n=4,101)
Unspecified football code (n=3,051)
Basketball (n=1,169)
Australian football (n=1,104)
Gymnastics (n=961)
Netball / handball (n=852)
Touch football (n=785)
Cricket (n=463)
Martial arts / wrestling (n=341)
Track and field (n=278)
Baseball / softball (n=226)
Field Hockey (n=203)
Tennis / raquet sports (n=155)
Equestrian (n=109)
0%
10%
20%
30%
40%
50%
60%
70%
80%
90%
100%
5-8 years
9-12 years
13-15 years
